# Ablation‐Immune Thermal Armor Via Microstructural‐Compatibility Design for Extreme Thermal Conditions

**DOI:** 10.1002/advs.77482

**Published:** 2026-08-31

**Authors:** Hongkang Ou, Lingxiang Guo, Bing Liu, Ziyi Yi, Yuyu Zhang, Jia Sun, Qiangang Fu

**Affiliations:** ^1^ Shaanxi Key Laboratory of Fiber Reinforced Light Composite Materials Northwestern Polytechnical University Xi'an People's Republic of China

**Keywords:** ablation resistance, plasma spraying, thermal protection, ultrahigh temperature ceramics

## Abstract

To overcome the brittleness and microstructural degradation of ultrahigh temperature ceramics (UHTCs) in hypersonic thermal protection systems, this work develops a C/C‐ZrC‐SiC/ZrC‐SiC composite via a microstructural‐compatibility design that integrates reactive melt infiltration with supersonic atmospheric plasma spraying. Critically, this work strategically incorporated SiC as a dispersed phase in the ZrC coating to actively regulate the deposition thermodynamics and oxidation kinetics, which suppresses the formation of continuous, lamellar ZrO_2_ interlayers and mitigates residual stress. Consequently, the designed composite demonstrates exceptional ablation resistance, withstanding oxyacetylene ablation (2200°C) for 3080 s and Ar‐H_2_ plasma ablation (2600°C) for 1500 s, while achieving an ultralow linear ablation rate on the order of 10^−5^ mm s^−1^. This work validates a microstructural‐compatibility‐led design principle, providing a foundational blueprint for developing thermal protection systems.

## Graphical abstract

This study unveils a microstructural‐compatibility design strategy that integrates reactive melt infiltration with plasma spraying to fabricate carbon fiber‐reinforced ZrC‐SiC composites. The deliberate incorporation of SiC into the ZrC coating releases heat accumulation, suppresses detrimental lamellar ZrO_2_ formation, and reduces residual stress. This approach endows the composite with exceptional ablation resistance, successfully achieving long‐term protection under extreme thermal environments exceeding 2600°C.

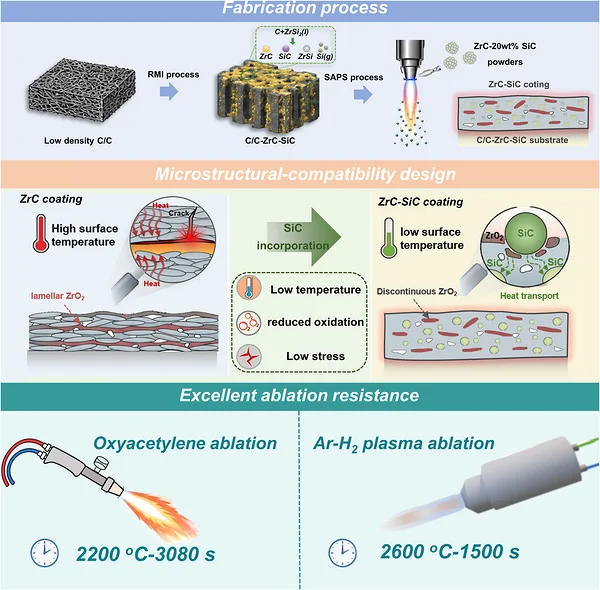

## 1 Introduction

Ultrahigh temperature ceramic (UHTC) materials, known for their exceptional high‐temperature performance in extreme environments, are considered key candidate materials for thermal protection systems in hypersonic vehicles [1, 2]. Among UHTCs, ZrC, ZrB_2_, TaC, high‐entropy carbides, and high‐entropy boride ceramic materials all exhibit excellent ablation resistance under extreme ablation conditions [3, 4, 5, 6, 7]. In particular, the ZrC‐SiC system exhibits outstanding comprehensive properties [8]. ZrC offers an extremely high melting point (∼3540°C) and excellent ablation resistance, forming a refractory zirconia (ZrO_2_) barrier upon oxidation. Meanwhile, SiC generates a continuous and dense silica (SiO_2_) glass layer when exposed to oxygen, which effectively inhibits oxygen diffusion and enables self‐healing of cracks [9]. However, the inherent brittleness and poor thermal shock resistance of these ceramics limit their reliable application under extreme environment and mechanical loads [10]. This represents the first universal challenge hindering their widespread engineering application.

To overcome the brittleness of ceramics, utilizing ZrC‐SiC ceramics for matrix modification of carbon fiber (C_f_) preforms is an effective strategy [11, 12]. The matrix‐modification methods mainly involve reactive melt infiltration (RMI) and precursor infiltration and pyrolysis (PIP) [13, 14, 15]. These approaches ingeniously utilize the toughening effect of carbon fibers to construct an interpenetrating “fiber‐ceramic” network within the material, successfully producing bulk materials that combine excellent toughness with excellent ablation resistance [16]. The carbon fiber‐reinforced ZrC‐SiC composites derived from the PIP method offer excellent mechanical properties, while exhibiting high cost and long processing time [17, 18]. In contrast, the RMI process is simple, yields highly dense composites, and is both time‐efficient and cost‐effective [19, 20]. However, the inherent characteristics of the RMI process often result in a material surface with microstructural inhomogeneity and residual low‐melting‐point phases [21]. This would lead to insufficient resistance against high‐speed gas flow erosion at the surface, forming key bottleneck limiting its application.

To address this surface protection limitation, introducing a coating on the Cf‐reinforced ceramics, such as depositing coatings via plasma spraying on the RMI‐derived C/C‐ZrC‐SiC composites, offers a promising route for constructing a dense and robust final defense layer [16, 22]. The plasma spraying method, with high temperature and high velocity, enables the preparation of thick coatings with low porosity and high bonding strength [23]. Nevertheless, critical problems emerged upon combining the surface coating and the matrix modification strategies. First, the interfacial compatibility between the plasma‐sprayed coating and the RMI‐modified substrate, including the matching of their coefficients of thermal expansion (CTE), chemical properties, and microstructure, directly determines the adhesion stability of the coating during thermal cycling. Second, the intrinsic relationship between the oxidation behavior of ZrC particles under the extreme processing conditions of plasma spraying, the coating microstructure (such as defects, phase distribution), and the thermal cycling resistance remains unclear. Moreover, conventional strategies attribute performance enhancement primarily to the compositional contribution of SiC, yet fail to recognize that the introduction of a dispersed second phase can fundamentally reshape the architecture of the coating during deposition, which could play an equally or even more dominant role in determining ablation resistance.

Consequently, based on interfacial compatibility design and microstructural control, this work proposed a high‐performance C/C‐ZrC‐SiC/ZrC‐SiC composite system. By investigating the microstructural evolution of the fabricated ZrC‐SiC coating, the atomic‐to‐microscale interfacial bonding mechanisms, and their deterministic role in ablation resistance, this work established the fundamental interplay between processing, microstructure, and performance in thermal protection coatings. The specific objectives were as follows: (i) to examine how adding SiC affects the microstructure, phases, and residual stress by controlling ZrC oxidation during spraying; (ii) to test ablation resistance in two extreme conditions (oxyacetylene and plasma flames) and link coating structure to its failure behavior. The closed‐loop logic of such microstructure‐compatibility‐led design was successfully verified since the C/C‐ZrC‐SiC/ZrC‐SiC system provided reliable protection under extreme conditions (oxyacetylene ablation: 2200°C‐3080 s; Ar‐H_2_ plasma ablation: 2600°C‐600 s). Furthermore, this fundamental understanding is pivotal for advancing the design and fabrication of next‐generation, high‐performance, and long‐lifetime thermal protection systems.

## 2 Results and Discussion

### 2.1 Microstructural Control of the Coatings

**FIGURE 1:**
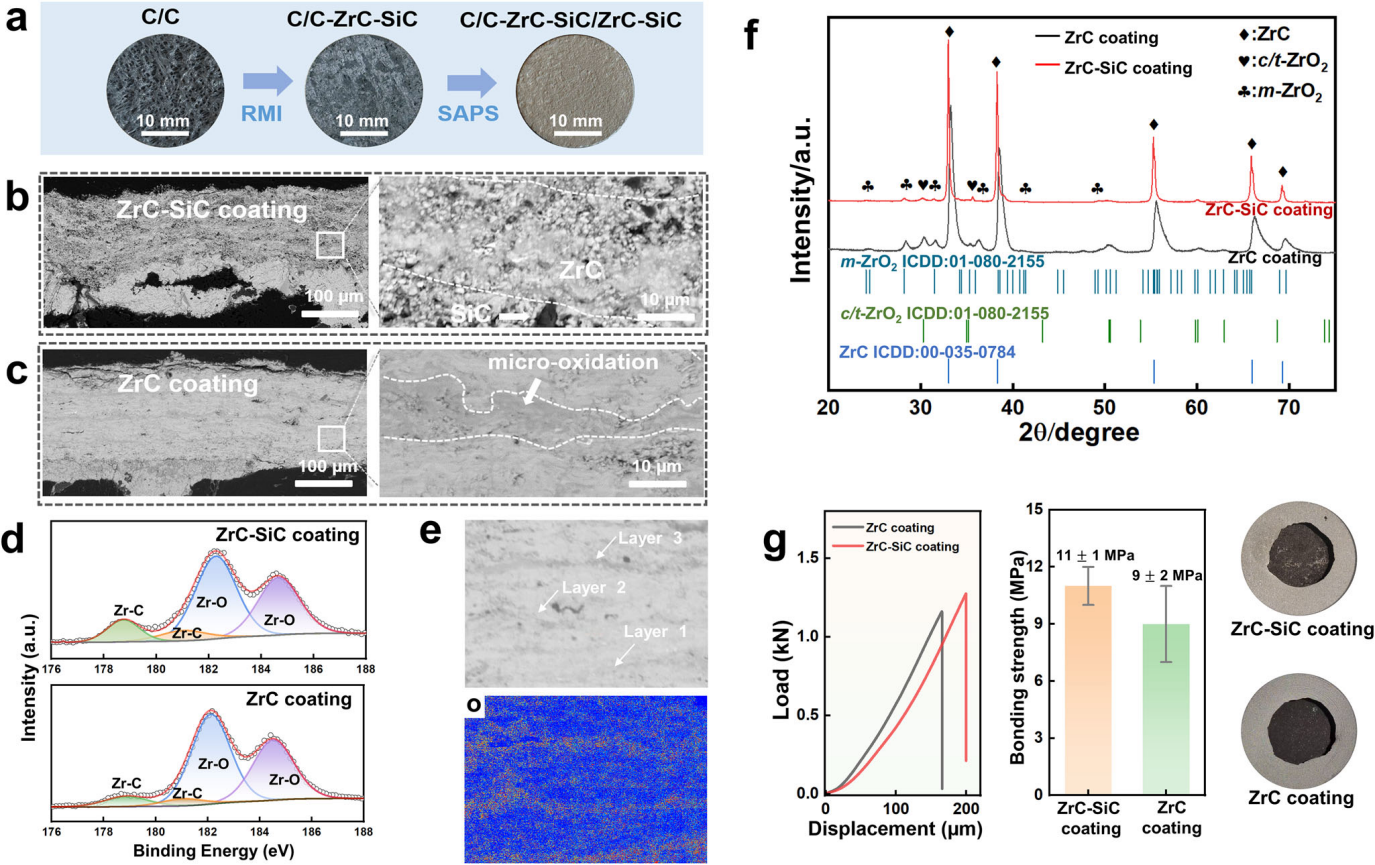
Characterizations of the as‐sprayed coatings: (a) surface pictures of the C/C sample after RMI and SAPS processes during fabrication; (b) cross‐section images of the ZrC‐SiC coating and its magnified image; (c) cross‐section images of the ZrCcoating and its magnified image; (d) XPS results corresponding to *Zr‐3d* peaks of the as‐sprayed coatings, where d^I^ and d^II^ display the XPS pattern of the ZrC‐SiC and ZrC coatings, respectively; (e) distribution maps of the O and C elements in the ZrC coating via EPMA method which proved the existence of the micro‐oxidation interlayer; (f) XRD patterns of the as‐sprayed coatings; (g) load‐displacement curves, bonding strength, and the fractographs of the as‐sprayed coatings, which exhibited higher bonding strength of the ZrC‐SiC coating.

A two‐step method, including reactive melt infiltration (RMI) and supersonic atmosphere plasma spraying (SAPS), was used to fabricate the C/C‐ZrC‐SiC/ZrC‐SiC sample. The optical images (Figure 1a) show that the porous C/C transferred to a black color, and they were densified to 3.1 g cm^−3^ after RMI. XRD patterns (Figure S1a) indicate that the surface phase composition consists of ZrC, SiC, and residual ZrSi_2_, which was consistent with the BSE image (Figure S1b). After thermal spraying, a ZrC‐based coating was deposited on the surface of the C/C‐ZrC‐SiC sample (in grey color as shown in Figure 1a) with a thickness of ∼200 µm. The molten droplet, after being heated by plasma, melted and deposited on the specimen surface. During the intervals between coating deposition cycles, ZrC was prone to oxidation when in contact with surrounding oxygen at high temperatures. Therefore, the grey phase in the ZrC coating (magnified cross‐section image in Figure 1c) indicated the micro‐oxidation of the ZrC droplets. Besides, the grey phase exhibited a lamellar structure, which could influence the integrity of the coating. The effects of SiC content on the microstructure and ablation performance of the coatings were compared, exhibiting that the coating with 20 wt.% SiC addition remained intact after ablation, whereas coatings with lower SiC content still exhibited spallation (Figure S2). Therefore, the subsequent research on ZrC‐SiC coatings adopted the addition of 20 wt.% SiC. Although a small amount of grey phase was observed in the ZrC‐SiC coating (Figure 1b), it did not exhibit a continuous layer‐featured distribution. Besides, the dispersed dark particles correspond to the SiC phase. During the spraying process, SiC would not melt but instead directly decompose and vaporize at high temperatures, resulting in dissipating a portion of the heat and leaving pores within the ZrC‐SiC coating [24]. XPS patterns show strong peaks corresponding to Zr─O bonds in both coatings, indicating the oxidation process of the ZrC during spraying (Figure 1d). The binding energy peaks of the *Zr‐3d* orbitals were normalized to obtain the percentage of the Zr─O chemical state using *A_Zr‐O_/A_Zr 3d_* (*A* represents area). The *A_Zr‐O_/A_Zr 3d_* was 0.89 in the ZrC coating, while it reduced to 0.82 in the ZrC‐SiC coating, indicating severe oxidation of the ZrC coating during spraying. Another EPMA analysis was conducted on the ZrC coating, and the distribution images revealed that the lamellar grey phase shows a high oxygen concentration, which provided evidence for micro‐oxidation (Figure 1e). XRD patterns also reveal that ZrC was the main phase in the as‐sprayed coating, while *m*‐ZrO_2_ and *c/t*‐ZrO_2_ emerged due to the micro‐oxidation (Figure 1f). Similarly, the peaks corresponding to the *m*‐ZrO_2_ and *c/t*‐ZrO_2_ in the XRD pattern of the ZrC coating were stronger than those in the ZrC‐SiC coating, further indicating the stronger oxidation of the ZrC coating, which was consistent with the XPS results. A tensile test was conducted on the coatings to test their bonding strength, and the load‐displacement curves were displayed in Figure 1g. The bonding strength of the ZrC‐SiC coating (11 ± 1 MPa) was slightly higher than that of the ZrC coating (9 ± 2 MPa). Based on the aforementioned analysis, the introduction of the SiC dispersed phase eliminated the layered micro‐oxidation structure, while simultaneously mitigating the oxidation degree of ZrC, thereby improving the compatibility between the ZrC‐based coating and the C/C‐ZrC‐SiC substrate.

**FIGURE 2:**
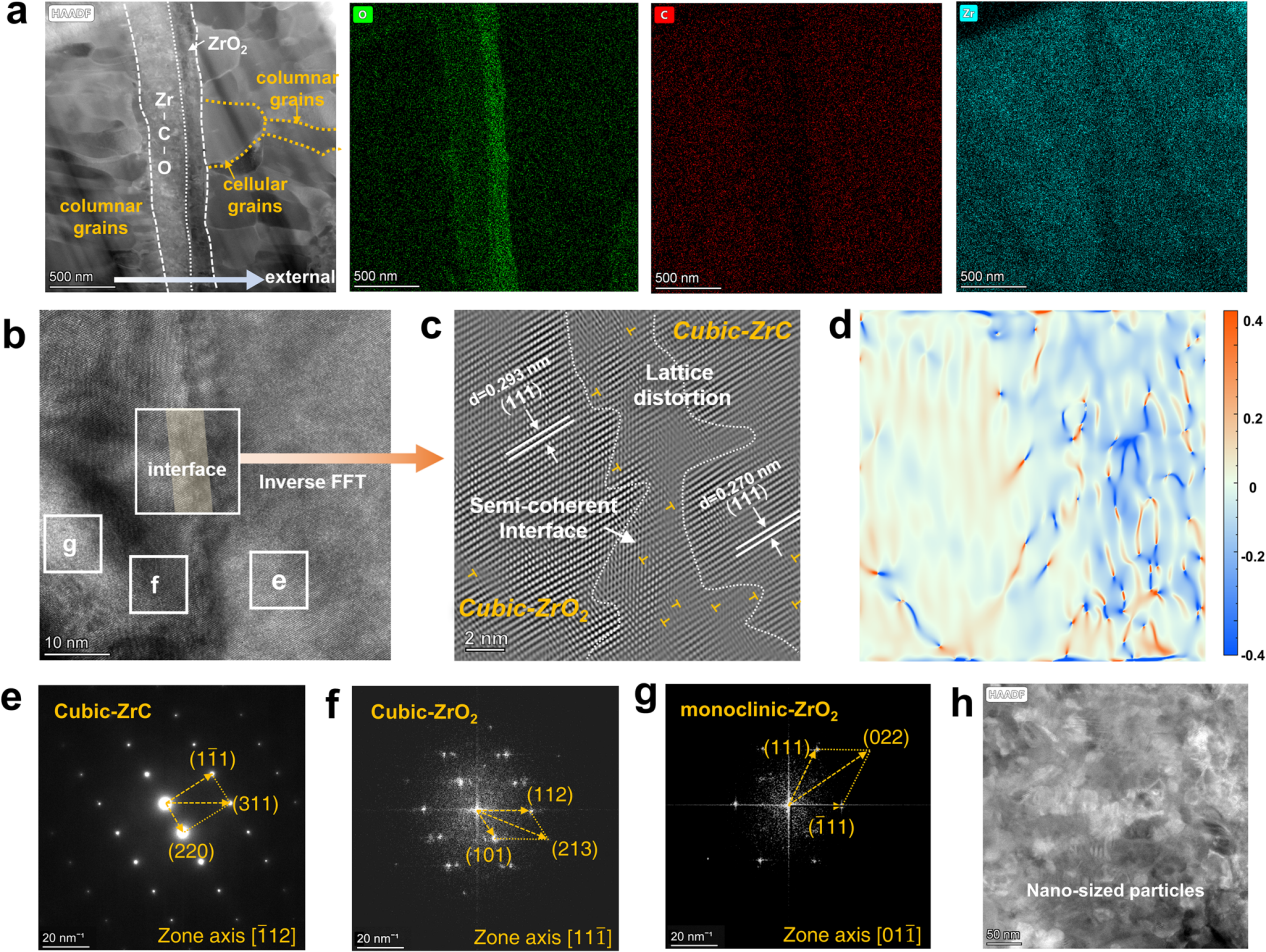
FIB‐TEM characterization of the interface between ZrC droplets: (a) the Zr‐O layer and Zr‐C‐O layer were observed from the HAADF image and its corresponding EDS maps of the ZrC droplets; (b) HRTEM image of the interface between ZrC and the ZrO_2_; (c) typical semi‐coherent interface could be found from the inverse FFT image corresponding to the magnified interface in (b); (d) large lattice strain formed at the interface from the GPA maps corresponding to (c); (e–g) *c‐*ZrC, *c‐*ZrO_2_, and *m‐*ZrO_2_ were identified from the fast Fourier transform analysis of the regions e, f, and g in (b); (h) magnified HAADF image corresponding to the Zr‐C‐O layer in (a).

Further investigation of the interfacial bonding state between ZrC droplets is essential to establish the correlation among atomic‐to‐microscale structure, stress state, and service performance under extreme thermal environments. Therefore, FIB‐TEM cross‐sectional analysis was performed on the droplet‐assembled ZrC coating to elucidate the bonding mechanism between the droplets (Figure 2). From the HAADF image in Figure 2a, the droplets formed nanoscale grains after cooling and a solidification process, unlike the environmental barrier coating (EBC, mainly silicate), which typically forms an amorphous phase [25]. Influenced by the temperature gradient from external air cooling, columnar grains developed, and cellular grains formed at the interface in contact with the prior droplets. Combined with the elemental distribution maps (Figure 2a), oxidation was observed at the interface among the droplets. After the solidification of the prior droplet, the outermost columnar grains contacted with oxygen, which was oxidized to form ZrO_2_ and lost its columnar structure. Beneath the ZrO_2_ layer, a Zr‐O‐C layer emerged, which was controlled by the content of oxygen since diffusion of oxygen was blocked by the ZrO_2_ layer. An amorphous‐to‐nanocrystalline transformation phenomenon occurred during the oxidation of carbides at low temperatures (below 1000°C) [26], resulting in discrete nanoscale grains. Therefore, the Zr‐C‐O layer contained a large amount of nanoscale grains from the magnified HAADF image in Figure 2h. The interfacial bonding mechanism between the ZrO_2_ and the newly deposited ZrC droplets was further investigated (Figure 2b,c). *c*‐ZrO_2_ was formed from the oxidation of the ZrC, and then *c*‐ZrC was deposited on this phase. The interface between the ZrO_2_ layer and the cellular ZrC grains exhibited a typical semi‐coherent interface, where dislocations and lattice distortions existed. Fundamentally, this was attributed to a slight mismatch in lattice parameters, since the interplanar spacing of the (111) face was 0.270 nm in *c*‐ZrC and 0.293 nm in *c*‐ZrO_2_. Furthermore, stress would concentrate on such a semi‐coherent interface (from the GPA image in Figure 2d), which might influence the stability of the coating during thermal cycles. In addition to the *c*‐ZrO_2_ and *c*‐ZrC phases corresponding to Figure 2f,g, the presence of *m*‐ZrO_2_ was also identified (region g of Figure 2b), which corroborates the XRD findings (Figure 2e). It was worth noting that ZrO_2_ and ZrC exhibit distinct thermophysical properties, particularly a significant difference in their coefficients of thermal expansion. This suggests that the presence of a ZrO_2_ interfacial layer may pose challenges to the thermal stability of ZrC‐based coatings.

### 2.2 Investigation on Thermophysical States of Coatings

**FIGURE 3:**
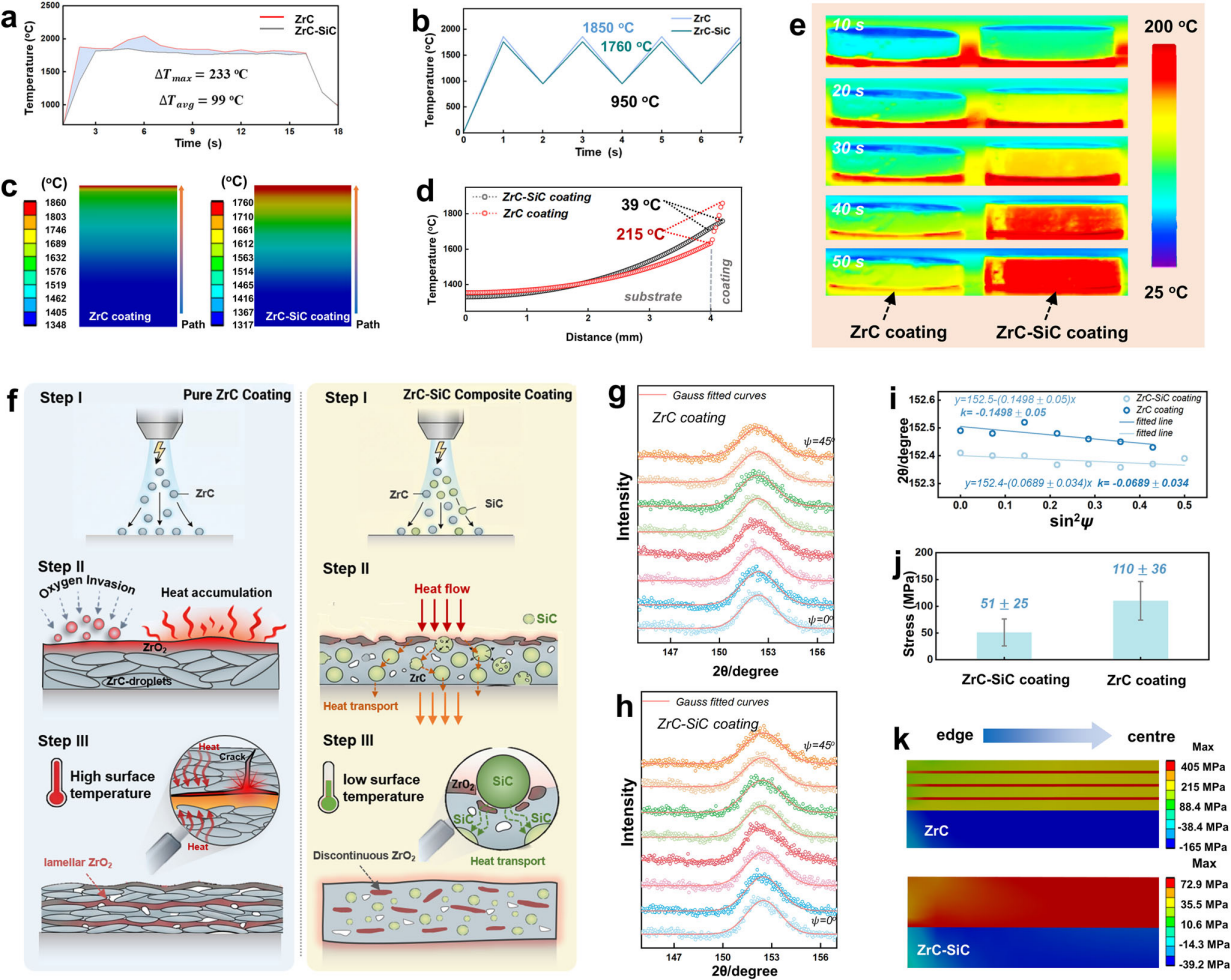
Residual stress analysis of the as‐sprayed coatings: (a) surface temperature of the coatings during continuous plasma spraying; (b) surface temperature setting of the coatings used in FEA models to simulate the repeated spraying; (c) temperature distribution of the samples with ZrC and ZrC‐SiC coatings after spraying; (d) temperature distribution of the samples with ZrC and ZrC‐SiC coatings after spraying along the path from the substrate to the coating; (e) infrared thermal images during the heating process of the samples with ZrC and ZrC‐SiC coating, respectively; (f) schematic diagram of the deposition mechanisms for ZrC and ZrC‐SiC coatings; (g, h) XRD patterns of the ZrC (g) and ZrC‐SiC (h) coatings with different angle of incidence ((531) plane of ZrC phase); (i) relationship between the sin^2^ *ψ* and the Gauss‐fitted center of the peaks; (j) the measured residual stress of ZrC and ZrC‐SiC coatings; (k) simulated thermal stress of the samples with ZrC and ZrC‐SiC coatings.

Owing to the extreme oxidation susceptibility of ZrC, it begins to undergo pulverizing oxidation at temperatures of 500°C, with the severity intensifying as temperature rises [27]. Therefore, the deposition temperature during coating preparation played a critical role in determining the microstructure and stress state of the coating. The surface temperature of both ZrC‐SiC and ZrC coatings was tested during the spraying process (Figure 3a). The result revealed that the maximum temperature of the ZrC coating reached 2046°C, and that of the ZrC‐SiC coating was 1850°C. The average deposition temperatures were 1850°C for the ZrC coating and 1760°C for the ZrC‐SiC coating, resulting in a maximum temperature difference of 233°C and an average difference of 99°C. The higher deposition temperature implied severe oxidation of ZrC, which was consistent with the phenomena observed in the aforementioned XRD and XPS results (Figure 1d–f). Subsequently, finite element models were established to simulate the heat transfer and thermal stress distribution in the single‐layer ZrC‐SiC coating and a multilayer ZrC coating (with ZrO_2_ interlayers). Regarding the surface temperature conditions of the coatings, the peak temperatures for ZrC and ZrC‐SiC were set at 1850°C and 1760°C during four consecutive deposition cycles, respectively (Figure 3b). Between deposition intervals, the coatings rapidly cooled to 950°C before being rapidly reheated to their peak temperatures in the subsequent cycle. Figure 3c,d presents the simulated temperature distribution within the samples and along the path at the final deposition cycles. In the ZrC coating, the highest temperatures were concentrated within the coating region, while the ZrC‐SiC coating showed significant heat conduction to the substrate, resulting in a more continuous and gradual temperature distribution. From Figure 3d, it was evident that the ZrC coating reached 1850°C, while the temperature at the interface dropped to 1635°C, exhibiting a significant thermal gradient of 215°C. This indicated substantial heat accumulation within the ZrC coating, impeding effective heat transfer to the substrate. For the ZrC‐SiC coating, the maximum temperature and the interface temperature were 1760°C and 1721°C, respectively, yielding a much smaller gradient of only 39°C. To verify the superior thermal transport capacity of the ZrC‐SiC coating compared to the ZrC coating, specimens coated with ZrC and ZrC‐SiC, respectively, were placed on a heating plate (set at 200°C), where the coated surface were kept in close contact with the plate (Figure 3e). The temperature of the sample with the ZrC‐SiC coating gradually increased, and the temperature of the substrate approached 200°C after 50 s, indicating efficient heat transfer into the substrate. In contrast, the substrate temperature of the ZrC‐coated sample remained significantly lower, confirming that heat was largely confined within the ZrC coating.

For the ZrC coating, its oxidation during deposition led to the formation of a thin ZrO_2_ interlayer. This ZrO_2_ layer, characterized by its low thermal conductivity, could act as a thermal barrier layer. In the subsequent deposition cycles, this layer impedes efficient heat transfer from the surface, causing a progressive increase in surface temperature (Figure 3f). This elevated temperature, in turn, could exacerbate the oxidation of the underlying ZrC. Consequently, a vicious cycle was established, including heat accumulation, intensified oxidation, and the growing oxide layer, which further promoted heat accumulation. In contrast, for the ZrC‐SiC coating, the presence of SiC, which possesses superior thermal conductivity, facilitated rapid heat dissipation throughout the coating [28]. Furthermore, the decomposition of SiC during the spraying process consumed additional energy, thereby reducing the overall deposition temperature. These combined effects resulted in milder oxidation of the ZrC droplets, and no significant continuous oxide layer could be observed within the ZrC‐SiC coating.

To determine the residual stress within the coatings, the residual stress of the coatings was verified via the XRD sin^2^ *ψ* technique and computed with the following Equations ((1), (2), (3)) [29, 30]

(1) $$\sigma_{\phi }=M\cdot k=-\frac{E}{2\left(1+\upsilon \right)}\;\cot\;\theta_{0}\frac{\pi }{180}\frac{\partial \left(2\theta \right)}{\partial \left(\mathrm{si}n^{2}\psi \right)}$$

(2) $$M=-\frac{E}{2\left(1+\upsilon \right)}\;\cot\;\theta_{0}\frac{\pi }{180}$$

(3) $$k=\frac{\partial \left(2\theta \right)}{\partial \left(\mathrm{si}n^{2}\psi \right)}\;$$

In Equation (2), *E* was 400 GPa, *ν* was 0.19, and *θ_0_* was 76.1° (2*θ_0_* = 152.3°, (531) plane of ZrC), yielding a calculated value of M = −739 MPa, while *k* was determined by the test results. The XRD patterns obtained at different incidence angles for the ZrC and ZrC‐SiC coatings were subjected to Gaussian fitting (Figure 3g,h). The fitted peak centers were then substituted into Equation (3), and the values of *k_ZrC_* and *k_ZrC‐SiC_* were calculated to be −0.1498 ± 0.05 and −0.0689 ± 0.034, respectively (Figure 3i). Then, the residual stress ($\sigma_{\phi }$) of the ZrC and ZrC‐SiC coating was calculated from formula (1), which were 110 ± 36 and 51 ± 25 MPa, respectively (Figure 3j).

The thermal stress during the cooling process was simulated for both coatings, and the results were presented in Figure 3e. For the ZrC coating, the presence of the thin interfacial ZrO_2_ layer resulted in the maximum tensile stress of ∼405 MPa in this oxide layer upon cooling. The maximum tensile stress within the ZrC layer reached ∼255 MPa. In contrast, the incorporation of SiC in the ZrC‐SiC coating led to the reduction of the overall thermal expansion coefficient and a more uniform temperature distribution. Consequently, the maximum tensile stress in this coating was only ∼73 MPa, significantly lower than that of the ZrC coating. Experimental and simulation results revealed a significantly higher residual stress in the ZrC coating compared to the ZrC‐SiC coating after thermal spraying. The simulation results represent the ideal stress state of the coating. However, in the as‐fabricated coatings, microcracks, pores, and the second phase would lead to stress relaxation. Therefore, the simulation results still show that the residual stress of the ZrC coating was significantly higher than that of the ZrC‐SiC coating, which was consistent with the experimental measurements. During thermal cycling, the semi‐coherent interface between ZrC and ZrO_2_, combined with localized stress concentration at this interface, makes the ZrC coating susceptible to delamination and spallation (see Figures S3a and S5a).

From a compositional perspective, the oxidation of SiC indeed generates a SiO_2_ glass phase, which is known to seal cracks and inhibit oxygen diffusion. However, this effect alone cannot explain why the pure ZrC coating failed catastrophically by delamination under identical ablation conditions (Figures S3a and S5a). From a microstructural perspective, the introduction of SiC led to two key alterations, which are largely independent of chemical characteristic during ablation: (i) a reduction in peak deposition temperature by 233°C (Figure 3a), suppressing the continuous lamellar ZrO_2_ formation that acts as a premature failure interface; (ii) a decrease in residual tensile stress from 110 ± 36 to 51 ± 25 MPa (Figure 3j), which directly mitigates driving force for spallation. Therefore, the introduction of SiC plays different roles in terms of composition and structure.

### 2.3 Validation of the C/C‐ZrC‐SiC/ZrC‐SiC Protection System Based on Enhanced Compatibility Design

To evaluate the thermal protection capability, the ZrC‐SiC coating and ZrC coating were subjected to the oxyacetylene and Ar‐H_2_ plasma ablation tests. The oxyacetylene flame provides a high heat flux (up to 4.18 MW m^−2^) with moderate gas velocity, primarily inducing thermal gradient‐driven oxidation [31]. In contrast, the Ar‐H_2_ plasma torch generates an ultrahigh‐temperature (≥ 2600°C), high‐velocity (supersonic) gas stream with low oxygen content, where mechanical erosion (shear force) and active species play dominant roles during ablation. Consequently, the absolute values of ablation duration and linear ablation rate obtained from these two methods are not directly comparable. Instead, they should be viewed as complementary validations under different failure mechanisms. Oxyacetylene testing assesses long‐term oxidation resistance and structural stability under thermal cycling, while plasma testing evaluates resistance to coupled thermal‐chemical‐mechanical erosion in high‐enthalpy flow.

**FIGURE 4:**
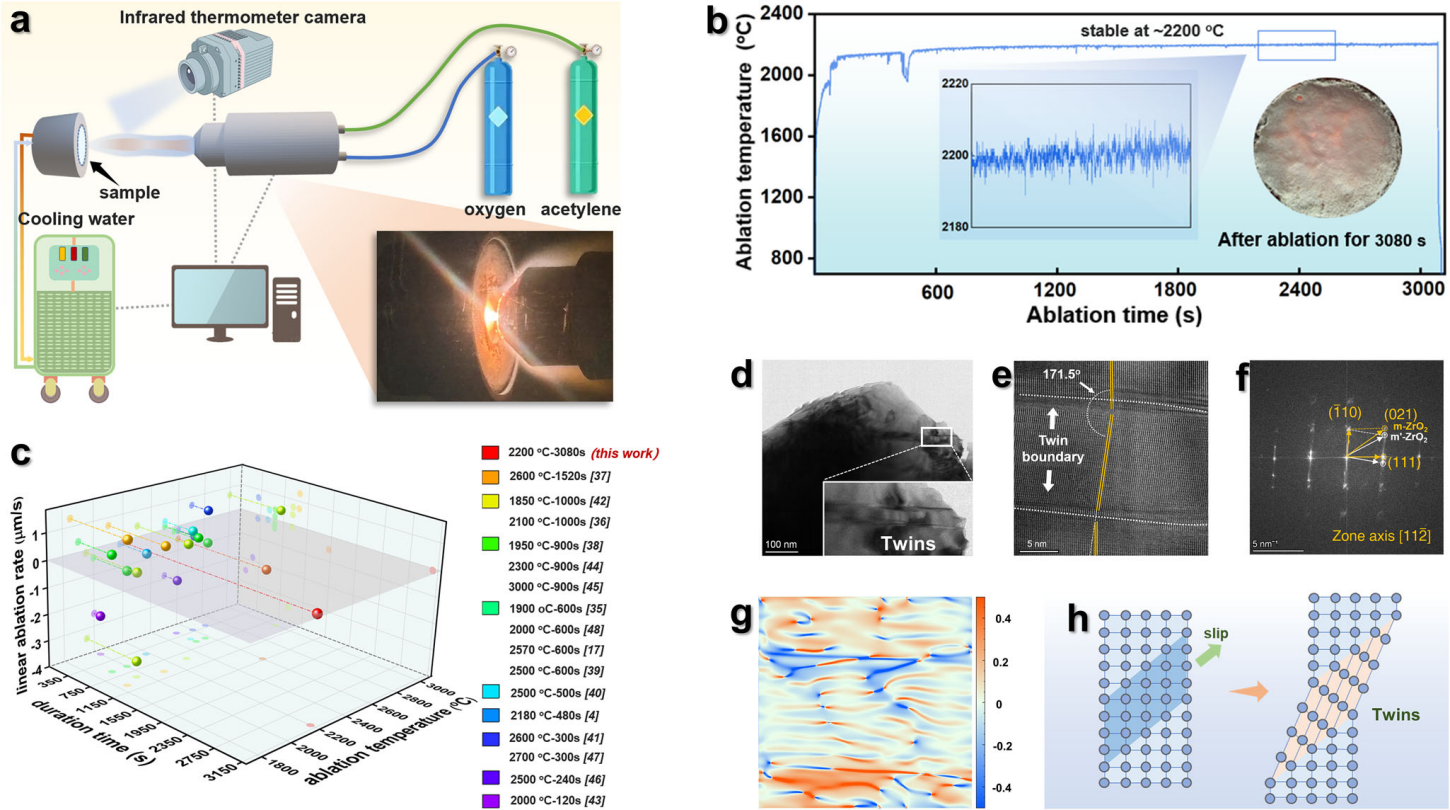
Oxyacetylene ablation behavior of the C/C‐ZrC‐SiC/ZrC‐SiC sample after exposure at 2200°C for 3080 s: (a) Schematic diagram of oxyacetylene ablation; (b) surface temperature curve and macroscopic images of C/C‐ZrC‐SiC/ZrC‐SiC sample after ablation; (c) comparison of ablation time and linear ablation rate of the thermal protection system after oxyacetylene ablation [4, 17, 35, 36, 37, 38, 39, 40, 41, 42, 43, 44, 45, 46, 47, 48]; (d) bright‐field image of the *m‐*ZrO_2_ via TEM; (e) HRTEM image of the *m‐*ZrO_2_ twinning; (f) FFT patterns corresponding to *m‐*ZrO_2_; (g) GPA image corresponding to (e); (h) schematic diagram of the of the *m‐*ZrO_2_ twinning transformation.

The ZrC‐SiC coating and ZrC coating were subjected to oxyacetylene ablation at 2.38 and 4.18 MW m^−2^ heat fluxes. As illustrated in Figure 4a, oxygen and acetylene were ignited as the reaction gases, and the flame impinged on the surface of the samples. After ablation at 4.18 MW m^−2^ for 240 s, the ZrC coating delaminated while the ZrC‐SiC coating remained intact (Figure S3a). The ablation temperatures were similar after 6 thermal cycles, and both reached ∼2640°C (Figure S3b). At this temperature, SiO_2_ underwent rapid volatilization and ZrO_2_ sintered into a dense structure, thereby enabling the ZrC‐SiC coating to form a dense ZrO_2_ outer layer (Figure S3c–f). Such results indicated the thermal protection capability of the C/C‐ZrC‐SiC/ZrC‐SiC system against oxyacetylene ablation at 4.18 MW m^−2^ heat flux. After oxyacetylene ablation at 2.38 MW m^−2^ heat flux for 1000 s (Figure S4a), only pores emerged on the surface of the ZrC‐SiC coating, while the ZrC coating still delaminated. The rapid temperature rise of the ZrC‐SiC coating during ablation indicated the swift formation of a protective scale, and the subsequent stable temperature profile demonstrated the maintained stability of this oxide layer under ablation conditions, manifesting excellent thermophysical compatibility between the ZrC‐SiC coating and the C/C‐ZrC‐SiC substrate. The linear ablation rate of the samples with ZrC coating and ZrC‐SiC coating was −1.22 × 10^−1^ and −6.01 × 10^−2^ µm s^−1^, respectively, revealing better ablation resistance of the C/C‐ZrC‐SiC/ZrC‐SiC system (Figure S4b). To further evaluate the stability of the C/C‐ZrC‐SiC/ZrC‐SiC system against ablation, the C/C‐ZrC‐SiC/ZrC‐SiC sample was tested up to 3080 s (Figure 4b). After ablation at 2200°C for 3080 s, the oxide scale remained intact, and the linear ablation rate was −7.5 × 10^−2^ µm s^−1^. From the XRD analysis (Figure S4c), ZrC transformed to *m*‐ZrO_2_ after ablation in all the samples, while SiC was oxidized to form SiO_2_ and subsequently volatilized at elevated temperatures. Figure 4c displayed a three‐dimensional plot of linear ablation rate versus ablation time and ablation temperature for the C/C‐ZrC‐SiC/ZrC‐SiC system compared with other thermal protection systems based on C/C under oxyacetylene torch ablation conditions. The C/C‐ZrC‐SiC/ZrC‐SiC system achieved an ablation duration of 3080 s in the oxyacetylene environment, which was one of the longest service lives reported to date. The *R_l_* reached an order of magnitude of 10^−2^ µm s^−1^, which was significantly lower than that of current protection systems (see Table S1 for more details). In summary, the C/C‐ZrC‐SiC/ZrC‐SiC system demonstrated substantial performance advantages over either single RMI modification or SAPS coating method.

The surface of the C/C‐ZrC‐SiC/ZrC‐SiC sample exhibited highly regular micro‐features embedded in ZrO_2_ grains (Figure S4d). A TEM analysis was conducted to identify such oxidation products. In the bright‐field image shown in Figure 4d, distinct twinning stripes could be observed, and the EDS analysis confirmed the presence of ZrO_2_ grains (Figure S4e). The HAADF‐STEM image (Figure 4e) and corresponding FFT pattern (Figure 4f) demonstrated the twins corresponding to *m*‐ZrO_2_. From the HAADF‐STEM image, typical intragranular twins emerged, accompanied by an 8.5° misorientation. During ablation, cubic ZrC transformed to *c/t*‐ZrO_2_, then it transformed to *m*‐ZrO_2_ during the cooling process with martensitic transformation as illustrated in Equations (4) and (5) [32]:

(4) $$ZrC+2O_{2}=ZrO_{2}+CO_{2}\left(g\right)$$

(5)

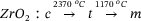




The FFT patterns also displayed two distinct sets of diffraction spots attributable to *m*‐ZrO_2_. Consistent with the characteristic deformation at twin interfaces, GPA (Figure 4g) quantification of *m*‐ZrO_2_ revealed pronounced lattice strain accompanied by sparse dislocation arrays along twin planes. Such lattice distortion induced by twinning martensitic transformation (Figure 4h) was demonstrated to contribute significantly to the toughening effect [4, 33]. At the same time, dislocations introduced by twinning could also contribute to strengthening and toughening in ceramics [34].

**FIGURE 5:**
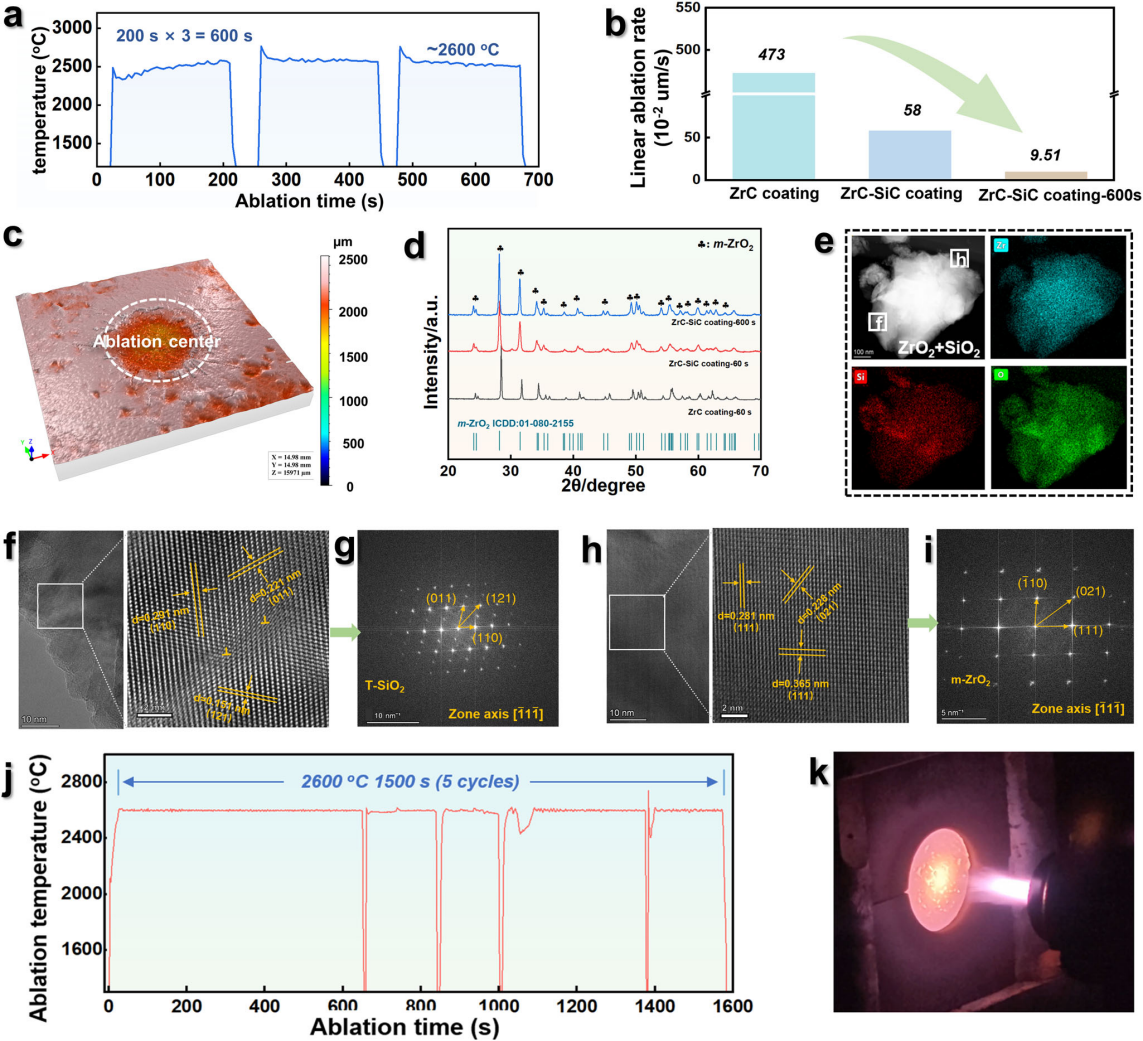
Ar‐H_2_ plasma ablation behavior of the C/C‐ZrC‐SiC/ZrC‐SiC sample after exposure at 2600°C for 600 s (200 s × 3): (a) surface temperature curve of C/C‐ZrC‐SiC/ZrC‐SiC during ablation; (b) linear ablation rate of the samples after ablation for 60 s and 600 s; (c) three‐dimensional surface topographical map of the C/C‐ZrC‐SiC/ZrC‐SiC sample after ablation; (d) XRD patterns of the ZrC and ZrC‐SiC coatings after plasma ablation; (e) HAADF image of the ZrO_2_‐SiO_2_ oxide; (f) HRTEM images corresponding to region f in (e); (g) fast fourier transform analysis demonstrated the presence of the SiO_2_; (h) HRTEM images corresponding to region h in (e); (i) fast fourier transform analysis demonstrated the presence of the ZrO_2_; (j) surface temperature curve of the C/C‐HfC‐SiC/HfC‐SiC system during plasma ablation at 2600°C upto 1500 s; (k) image of the C/C‐HfC‐SiC/HfC‐SiC sample during long‐term plasma ablation.

To further evaluate the protective performance of the samples under higher‐temperature and higher‐erosion‐speed conditions, both samples were subjected to Ar‐H_2_ plasma ablation [31]. After plasma ablation for 60 s (Figure S5a), the ZrC coating delaminated while the ZrC‐SiC coating remained intact. From the cross‐sectional BSE image of the samples (Figure S5b,c), the ZrC coating transformed into tiny ZrO_2_ grains with a loose structure. The ZrC‐SiC coating transformed to the ZrO_2_ and SiO_2_ phases with an interlocking structure due to the viscoelastic rheological effect of SiO_2_ at elevated temperature [49]. Due to the spallation of the ZrC coating, the surface roughness of the ZrC coating was significantly higher than that of the ZrC‐SiC coating after plasma ablation (Figure S5d,e). After 60 s of ablation, the linear ablation rate of the ZrC coating was 4.73 µm s^−1^, while that of the ZrC‐SiC coating was only 5.8 × 10^−1^ µm s^−1^, which was merely 12% of the ZrC coating (Figure 5b). A longer plasma ablation test (200 s per cycle, 600 s in total) was conducted on the C/C‐ZrC‐SiC/ZrC‐SiC system, and the response temperature is displayed in Figure 5a. At the beginning of each cycle, the temperature showed a sudden rise due to the accumulation of heat. Then, the coating dissipated heat effectively, and the temperature was maintained at ∼2400°C. The response temperature gradually increased to ∼2600°C with prolonged ablation time to 200 s, and stabilized at 2600°C in the next cycles up to 600 s. Figure S5f shows the optical image and the surface morphologies of the sample after plasma ablation, and an ablation pit could be observed on the surface. Once the ZrO_2_ protective layer was eroded, the exposed SiC and ZrC oxidized to form SiO_2_ and ZrO_2_. In the ablation centre of the surface (Figure S5f ^I^), ZrO_2_ skeleton and SiO_2_ could be observed, which manifested that the carbon fibers were still protected by the oxide scale. Due to the significant reduction in viscosity of SiO_2_ at high temperatures, it flowed away or volatilized under the gas flow, leaving the ZrO_2_ skeleton [49]. In non‐ablated regions (Figure S5f ^II^), the decreased temperature allowed SiO_2_ to fill the intergranular spaces of ZrO_2_ (Figure S5g) [49]. The *R_l_* of the C/C‐ZrC‐SiC/ZrC‐SiC sample after plasma ablation for 600 s was 9.51 × 10^−2^ µm s^−1^ (Figure 5b), exhibiting excellent ablation resistance. At the edge area of the ablation pit (Figure 5c), typical dynamically eroded surface morphology could be seen (photographic documentation of the ablation process at various stages was provided in movies S1–S3). With a melting point of 2650°C–2700°C, ZrO_2_ underwent rapid softening when ablation temperatures exceeded 2600°C, leading to spallation under intense aerodynamic shear forces.

XRD analysis was used to identify the phases on the surface after ablation. Only *m*‐ZrO_2_ could be detected on the surface of the sample after plasma ablation, which was similar to the oxyacetylene ablation (Figure 5d). A TEM analysis was conducted to study the composition of region b^II^ in Figure 5b, and the grain consisted of the Si, Zr, and O elements, which could be ZrO_2_ and SiO_2_ (Figure 5e). From the HAADF‐STEM image and corresponding FFT pattern (Figure 5f,g, region f in Figure 5e), the d‐spacings were measured to be 0.291 and 0.221 nm, corresponding to (110) and (011) planes along [$\bar{1}1\bar{1}$] orientation of the SiO_2_ phase, respectively. Moreover, Figure 5h,i validated the formation of *m*‐ZrO_2_, with typical ($\bar{1}10$) and (111) planes (d‐spacings of 0.365 and 0.281 nm, respectively) along the [$\bar{1}1\bar{1}$] direction. Despite the reaction between ZrO_2_ and SiO_2_ forming ZrSiO_4_, ZrSiO_4_ underwent destabilization and decomposition above 1700°C. Consequently, TEM characterization definitively confirmed the presence of the ZrO_2_ and SiO_2_ phases with an interlocked structure.

To validate the interface compatibility enhancement strategy while improving the ablation resistance temperature of the oxide scale, we adopted the HfC‐SiC coating system, utilizing the high melting point of HfO_2_ to raise the softening temperature of the oxide scale and thereby enhance the protective performance of the coating under Ar‐H_2_ plasma ablation at 2600°C. HfC has a relatively higher density than ZrC, thus, 10 wt.% SiC was added to balance the volume fraction. The cross‐sectional SEM images and XRD patterns of the relevant coatings are provided in Figure S6. The C/C‐HfC‐SiC/HfC‐SiC protection system, developed using a comparable strategy to overcome the limitations of ZrO_2_ above 2600°C, successfully withstood Ar‐H_2_ plasma ablation at 2600°C for 1500 s (Figure 5j,k and Movie S4). After thermal cycles and long‐term ablation, the formation of the HfO_2_ protective scale prevented the underlying carbon fibers from ablation or oxidation due to the higher melting point, confirming the excellent performance of such a compatibility tailoring strategy.

### 2.4 Investigation on the Structural Evolution of the C/C‐ZrC‐SiC/ZrC‐SiC Sample

**FIGURE 6:**
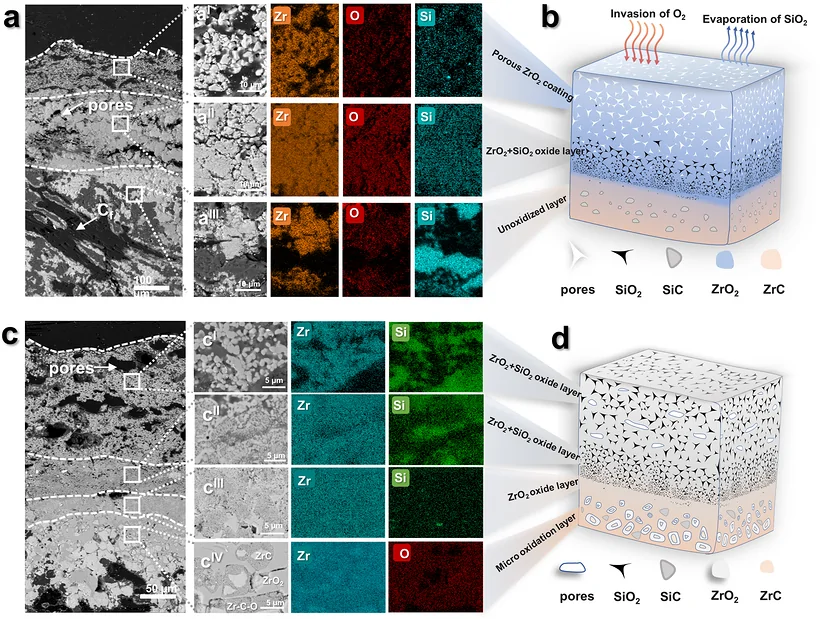
Cross section images and schematic diagrams illustrating the microstructure evolution of the C/C‐ZrC‐SiC/ZrC‐SiC sample after oxyacetylene and plasma ablation: (a) cross section images of the C/C‐ZrC‐SiC/ZrC‐SiC sample after oxyacetylene ablation for 3080 s, where a^I^, a^II^, a^III^ show the top of the coating, the surface ceramic layer of the C/C‐ZrC‐SiC substrate, and the interior of the C/C‐ZrC‐SiC composite, respectively, acompanied by their corresponding EDS maps; (b) schematic diagram illustrating the microstructure evolution of the C/C‐ZrC‐SiC/ZrC‐SiC sample after oxyacetylene ablation; (c) cross section images of the C/C‐ZrC‐SiC/ZrC‐SiC sample after Ar‐H_2_ plasma ablation for 600 s, where c^I^, c^II^, c^III^, and c^IV^ show the top of the coating, the bottom of the coating, the surface ceramic layer of the C/C‐ZrC‐SiC, and the interior of the C/C‐ZrC‐SiC composite, respectively, accompanied by their corresponding EDS maps; (d) schematic diagram illustrating the microstructure evolution of the C/C‐ZrC‐SiC/ZrC‐SiC sample after Ar‐H_2_ ablation.

The cross‐section of the C/C‐ZrC‐SiC/ZrC‐SiC sample after ablation was characterized to analyze the phase and microstructural evolution after long‐duration exposure. Figure 6a ^I^, a^II^, and a^III^ show the cross‐sections of the ZrC‐SiC coating, the surface ceramic layer of the C/C‐ZrC‐SiC substrate, and the interior of the C/C‐ZrC‐SiC composite, respectively, after oxyacetylene ablation at 2200°C for 3080 s. During the initial ablation stage, the SiO_2_ formed from the oxidation of SiC could effectively fill microcracks. However, as ablation time increased, the SiO_2_ gradually volatilized, resulting in the formation of a porous ZrO_2_ outer layer in the ZrC‐SiC coating (Figure 6a ^I^). Prolonged oxygen attack led to the oxidation of the carbon fibers, leaving numerous pores within the ceramic phase within ∼200 µm thickness on the surface region of the C/C‐ZrC‐SiC substrate. The ceramic layer formed after RMI remained dense after oxidation and did not develop a porous structure. However, due to the limited volatilization of SiO_2_ under the protection of the outer coating, which originated from the presence of residual Zr‐Si compounds after RMI and the subsequent oxidation, there existed some porosity in this region (Figure 6a ^II^) [50]. In the interior region of the C/C‐ZrC‐SiC substrate (Figure 6a ^III^), oxygen infiltration was effectively mitigated. As indicated by the EDS analysis, ZrC underwent only slight oxidation, while SiC remained largely unoxidized, and the carbon fibers showed no signs of oxygen attack. Although the porous structure formed in the ZrC‐SiC coating would reduce its oxygen‐blocking capability, the ceramic layer within the C/C‐ZrC‐SiC substrate itself retained considerable resistance to oxygen diffusion by continuously forming SiO_2_. This ensured the protection of the internal carbon fibers from oxidation, which was crucial for maintaining the mechanical properties and extending their ablation lifetime.

The schematic diagram illustrates the ablation mechanism of the C/C‐ZrC‐SiC/ZrC‐SiC sample (Figure 6b). After prolonged ablation, the sample developed a multi‐layered structure from the exterior to the interior: a porous ZrO_2_ outer layer, an oxygen‐resistant layer composed of ZrO_2_ and SiO_2_ formed by the oxidation of the ceramic layer of the C/C‐ZrC‐SiC, and the C/C‐ZrC‐SiC substrate, where the internal ZrC phase was only slightly oxidized. The microstructural evolution of the C/C‐ZrC‐SiC/ZrC‐SiC sample was largely governed by the volatilization of SiO_2_. As the SiO_2_ continuously vaporized in the surface layer, residual silicon within the substrate would subsequently oxidize, forming new SiO_2_ that filled cracks and pores. Due to the thermal insulation provided by the outer coating layers, the temperature within the specimen gradually decreased, leading to a corresponding reduction in the volatilization rate of the internally formed SiO_2_. Due to the high oxygen content of the oxyacetylene flame, SiO_2_ continuously volatilizes during long‐term ablation, and oxygen permeation leads to gradual oxidation of the inner ceramic layer. Benefiting from the advantages of the ZrC‐SiC system, the inner Si element continuously generated SiO_2_ to sustain its oxygen‐barrier effect.

Unlike oxyacetylene ablation, Ar‐H_2_ plasma ablation features highly concentrated energy and high‐velocity gas flow scouring, making this environment more representative of the aerodynamic heating and shear conditions experienced by the nose, wing leading edges, and other critical parts of actual hypersonic vehicles. The cross‐sectional SEM image of the ablation center of the sample (Figure S7a) revealed that the ZrC‐SiC coating experienced severe erosion within the plasma jet, leading to complete coating removal, as schematically illustrated (Figure S7b). Under the severe scouring and the volatilization of a substantial amount of SiO_2_, the protective ZrO_2_‐SiO_2_ scale from the oxidation of the ZrC‐SiC coating struggled to maintain its phase and structural stability.

To elucidate the phase and structural evolution of the ZrC‐SiC coating during plasma ablation, characterization was performed specifically in the near‐center ablation region (Figure 6c,d). Figure 6c ^I^, c^II^, c^III^, and c^IV^ show the ablated cross‐sections and their respective elemental distribution maps of the ZrC‐SiC coating, the inner region of the ZrC‐SiC coating, the outer zone of the C/C‐ZrC‐SiC ceramic layer, and the inner zone of the C/C‐ZrC‐SiC ceramic layer, respectively. Within the ZrO_2_‐SiO_2_ protective layer formed by the oxidation of the ZrC‐SiC coating, the volatilization of SiO_2_ left large pores (Figure 6c ^I^). The pores within the ZrO_2_ skeleton could be healed by residual SiO_2_, which had not fully volatilized after ablation for 600 s, thus maintaining the oxygen diffusion barrier effect. Due to this sustained oxygen barrier capability, the inner portion of the ZrC‐SiC coating retained finer ZrO_2_ grains after oxidation compared to the outer region (Figure 6c ^II^), with the pores filled by SiO_2_. The physical interface between the sprayed coating and the underlying C/C‐ZrC‐SiC ceramic layer became almost indistinguishable, with the respective regions identifiable based on their phase and structural characteristics. In the outer zone of the C/C‐ZrC‐SiC ceramic layer (Figure 6c ^III^), ZrO_2_ grains were formed due to micro‐oxidation. The oxygen penetration depth within the C/C‐ZrC‐SiC ceramic layer was limited to merely 30–50 µm, attributed to the effective oxygen blocking by the outer coating layers and the oxygen‐depleted environment of the Ar‐H_2_ plasma flame. In the innermost region Figure 6c ^IV^), ZrC experienced slight oxidation. Elemental distribution maps confirmed that the ZrC was not fully oxidized (Figure S7c). The ZrC grains were surrounded by a Zr‐C‐O phase, forming a core‐shell structure. As the ablation time increased, oxygen diffused through this Zr‐C‐O phase, gradually oxidizing the inner ZrC core to Zr‐C‐O, while the outer Zr‐C‐O shell was further oxidized to ZrO_2_. This sequential oxidation process was primarily controlled by the diffusion of oxygen.

## 3 Conclusion

This work validates a microstructural‐compatibility design to address the intrinsic brittleness and microstructural degradation of ultrahigh temperature ceramics (UHTCs) for hypersonic thermal protection. By integrating reactive melt infiltration (RMI) and supersonic atmospheric plasma spraying (SAPS), we fabricated the C/C‐ZrC‐SiC/ZrC‐SiC composite, with SiC strategically incorporated as a dispersed phase in the ZrC coating to regulate deposition thermodynamics and oxidation kinetics.

The deposition temperature was effectively reduced (Δ *T_MAX_* = 233°C) through the incorporation of the SiC dispersed phase, thereby avoiding the vicious cycle triggered by surface heat accumulation and the formation of lamellar oxide layers. Through both simulation and experimental verification, the ZrC‐SiC coating exhibited a residual stress of 51 ± 25 MPa after compatibility design, which was 54% lower than the 110 ± 36 MPa measured for the ZrC coating. This designed microstructure enabled excellent structural stability during extreme thermal exposure by in‐situ forming a multi‐layered oxide scale. The synergistic effect of a porous ZrO_2_ skeleton and a crack‐sealing SiO_2_ glass phase provided a robust and self‐healing oxygen barrier. Consequently, the system withstood oxyacetylene ablation at 2200°C for an unprecedented 3080 s and Ar‐H_2_ plasma ablation at 2600°C for 600 s, achieving an ultralow linear ablation rate on the order of 10^−2^ µm s^−1^. This study establishes a generalizable design principle—linking targeted microstructural control to ablation resistance, providing a foundational blueprint for high‐performance thermal protection systems in extreme environments.

## 4 Experimental Methods

### 4.1 Sample Fabrication

The 2.5D needle‐punched carbon fiber preform was fabricated from PAN‐based carbon fiber (T300). Subsequently, the pyrolytic carbon (PyC) matrix was deposited in the fiber preform using the CVI method through the pyrolysis of CH_4_. Carbon/Carbon composites (C/C) with a density of 1.2–1.4 g cm^−3^ were used for subsequent processing. The C/C‐ZrC‐SiC/ZrC‐SiC samples were fabricated via a two‐step method, including the reactive melt infiltration (RMI) and the supersonic atmosphere plasma spraying (SAPS). For the RMI process, the ZrSi_2_ powders (particle size 10–20 µm, 99% purity, Jinzhou Shengwen New Material Co., Ltd., Jinzhou, China) were placed in a graphite crucible, and the C/C was embedded with the ZrSi_2_ powders [50]. After 1–3 h heat treatment at 1700°C–1900°C, the melten ZrSi_2_ powder infiltrated into the pores of C/C and reacted with PyC to form ZrC and SiC from Equation (6):

(6) $$C+ZrSi_{2}\left(l\right)\to ZrC+SiC+ZrSi+Si\left(g\right)$$

After the RMI process, the C/C‐ZrC‐SiC samples were ultrasonically cleaned for 30 min and dried at 80°C for 10 h to remove adherent particulates from the surface. Before thermal spraying, ZrC (1–3 µm, 99% purity, Jinzhou Shengwen New Material Co., Ltd., Jinzhou, China) powders with spherical shape were obtained through the spray‐drying method (centrifugal spray dryer, LGZ‐5, Wuxi, China). During thermal spraying, the ZrC powders were heated and melten by the plasma beam stream, then deposited onto the C/C‐ZrC‐SiC surface to form a ZrC coating. To improve the interfacial compatibility, the ZrC containing 20 wt. % SiC (1‐3 µm, 99% purity, Jinzhou Shengwen New Material Co., Ltd., Jinzhou, China) coating was fabricated through the above processes. Details of the SAPS process were summarized in Table S2.

### 4.2 Ablation Tests

The thermal protection performance of C/C‐ZrC‐SiC/ZrC‐SiC composites was evaluated through oxyacetylene torch ablation and plasma ablation tests. The oxyacetylene ablation tests were performed under an oxyacetylene torch (OA‐III) with heat flux of 4.18 and 2.38 MW m^−2^ according to GJB 323A‐96. The surface response temperature was detected from the short‐wave infrared thermography thermal imager (IR Thermography, PYROVIEW 768 N, Dresden, Germany). The distance between the nozzle and the surface was adjusted to 10 and 31 mm for the ablation tests with high and low heat flux, respectively. For the high heat flux (4.18 MW m^−2^) ablation test, the pressure and flow rates of C_2_H_2_ were 0.095 MPa and 0.31 L s^−1^, and 0.40 MPa and 0.42 L s^−1^ for O_2_. Both C/C‐ZrC‐SiC/ZrC and C/C‐ZrC‐SiC/ZrC‐SiC samples were exposed to the flame for 240 s. For the low heat flux (2.38 MW m^−2^) ablation test, the pressure and flow rates of C_2_H_2_ were 0.095 MPa and 0.18 L s^−1^, and 0.4 MPa and 0.24 L s^−1^ for O_2_. The 1000s sustained ablation testing was conducted on both C/C‐ZrC‐SiC/ZrC and C/C‐ZrC‐SiC/ZrC‐SiC samples to evaluate long‐term thermal stability under extreme conditions. After that, the C/C‐ZrC‐SiC/ZrC‐SiC samples were further subjected to the flame for up to 3080 s.

The plasma ablation tests were conducted under the plasma torch system (HEPJ‐100, Beijing Academy of Armored Force Engineering), equipped with a two‐colored infrared pyrometer (STRONG‐SR‐7032, SIJIE OPTOELECTRONICS TECHNOLOGY Co., Ltd., China) to record the surface temperature. Ar and H_2_ were used as plasma‐generating gases; the gas pressure of the plasma flame was 3.0 MPa, and the fluxes of Ar and H_2_ were 75.0 L min^−1^ and 1.6 L min^−1^, respectively. The distance between the sample surface and the nozzle tip was controlled at 40 mm, and the power of the plasma torch was set to 35 kW. Both C/C‐ZrC‐SiC/ZrC and C/C‐ZrC‐SiC/ZrC‐SiC samples were exposed to the plasma flame for 60 s. After that, the C/C‐ZrC‐SiC/ZrC‐SiC sample was further tested to 3 × 200 s ablation cycles. The thickness of the sample center was measured before and after the ablation test, and the linear ablation rates (*R_l_*) were calculated according to Equation (7):

(7) $$R_{l}=\left(l_{0}-l_{t}\right)/t$$

*where l_0_* and *l_t_* are the thicknesses of the samples before and after ablation, respectively; t is the ablation time (s).

### 4.3 Characterizations

The microstructures and chemical compositions of the coatings were analyzed by a scanning electron microscope (SEM, JSM6460, Japan; ZEISS EVO10, Germany) at a voltage of 20 kV equipped with energy dispersive spectroscopy (EDS, Oxford INCA, Holland). The phase compositions of the coatings were characterized by x‐ray diffraction (XRD, X′Pert Pro MPD, PANalytical, Netherlands) with Cu Kα radiation at a step size of 0.01°, and 1s dwell counting time. The residual stress in the sprayed coating was measured using the x‐ray diffraction sin^2^ψ technique. An in situ lift‐out technique using a focused ion beam microscope (Helios G4 CX, Thermo Fisher Scientific, USA). Thin lamellae were machined by focused ion beam (FIB, FEI Helios 660) in the area where the ZrC droplets overlapped. The microstructure and element distribution of coatings before and after ablation were characterized by transmission electron microscopy (TEM, FEI Talos F200X, USA) at an accelerated voltage of 200 kV. The elemental compositions and local bonding environment of the respective elements of the as‐sprayed coatings were determined by x‐ray photoelectron spectroscopy (XPS, Axis Supra, Kratos, UK). The tensile tests were conducted by an electronic universal material testing machine (INSTRON 3382, USA) to test the bonding strength of the coatings with a tensile speed of 0.5 mm min^−1^. The cross‐sectional elemental distribution of the coating after spraying was analyzed by wavelength dispersive spectroscopy in an electron probe microanalyzer (EPMA‐8050G, Shimadzu, Japan), which allows the analysis of light elements like C and O. To accurately measure the surface temperature of the samples during the spraying process, a thermal test platform was established. In this setup, an infrared pyrometer was used to monitor real‑time temperature changes on the sample surface, while a plasma torch generated a high‑temperature plasma jet that carried the heated and molten powders to the sample surface, thereby depositing the coating.

### 4.4 FEA Results

A simplified Finite Element Analysis (FEA) model was established by the commercial package Ansys Workbench 2022 R2 to estimate the thermal stress after thermal spraying. The calculation of thermal stress was conducted within the range of linear elasticity. Thermophysical properties of relevant materials during simulation are listed in Table S3, where *ρ, α, λ, E, C*, and *v* represent the density, CTE, thermal conductivity, Elastic modulus, specific heat capacity, and Poisson's ratio, respectively.

## Author Contributions

**Hongkang Ou**: conceptualization, investigation, writing – original draft, methodology. **Lingxiang Guo**: methodology. **Bing Liu**: methodology. **Ziyi Yi**: methodology. **Yuyu Zhang**: methodology. **Jia Sun**: supervision, writing – review and editing. **Qiangang Fu**: supervision, resources, writing – review and editing.

## Funding

The research was supported by the National Natural Science Foundation of China (52125203, 52472107) and the National Defense Basic Scientific Research Program of China (JCKY2022607C007, JCKYS20250106).

## Conflicts of Interest

The authors declare no conflicts of interest.

## Supporting information




**Supporting File 1**: advs77482‐sup‐0001‐SuppMat.docx.

**Supporting File 2**: advs77482‐sup‐0002‐VideoS1.mp4.

**Supporting File 3**: advs77482‐sup‐0003‐VideoS2.mp4.

**Supporting File 4**: advs77482‐sup‐0004‐VideoS3.mp4.

**Supporting File 5**: advs77482‐sup‐0005‐VideoS4.mp4.

## Data Availability

The data that support the findings of this study are available from the corresponding author upon reasonable request.
