# Supplementary material for: Ablation‐Immune Thermal Armor Via Microstructural‐Compatibility Design for Extreme Thermal Conditions
Source: Adv Sci (Weinh). 2026 Aug 31:e77482. Online ahead of print. doi: 10.1002/advs.77482 (PMC13530017; doi:10.1002/advs.77482)
Supplement: Supplementary file 1 [file ADVS-9999-e77482-s002.docx]

Supporting Information

**Ablation-immune thermal armor via microstructural-compatibility design for extreme thermal conditions**

Hongkang Ou, Lingxiang Guo, Bing Liu, Ziyi Yi, Jia Sun *, Qiangang Fu *

Shaanxi Key Laboratory of Fiber Reinforced Light Composite Materials, Northwestern Polytechnical University, Xi’an, 710072, PR China

E-mail:<j.sun@nwpu.edu.cn> (J. Sun), <fuqiangang@nwpu.edu.cn> (Q.G. Fu)

Figure S1. Characterization of the C/C-ZrC-SiC sample after RMI, which exhibited the existence of the residual ZrSi_2_ phase: (a) XRD pattern; (b) the cross-section BSE image.

Figure S2. Characterization of the coatings with different SiC addition: (a) SEM images of the as-prepared coatings; (b) abaltion results of the coatings, where the ZrC, ZrC-3 wt.%SiC coatings delaminated while ZrC-10 wt.%SiC, and ZrC-20 wt.%SiC coatings kept relatively intact; (c) XRD patterns of the as-fabricated coatings; (d) XRD patterns of the coatings after ablation.

Figure S3. Characterization of the samples with ZrC and ZrC-SiC coatings after oxyacetylene ablation at 4.18 MW m^-2^ for 240 s: (a) surface and their 3D surface topography images; (b) surface temperature curves during ablation; (c) cross section images of the ZrC coating after ablation, where the c^I^ and c^II^ display the top and the interface of the coating; (d) surface image of the ZrC coating after ablation; (e) cross section images of the ZrC-SiC coating after ablation, where the e^I^ and e^II^ display the top and the interface of the coating; (f) surface image of the ZrC-SiC coating after ablation.

Figure S4. Characterization of the samples with ZrC and ZrC-SiC coatings after oxyacetylene ablation at 2.4 MW m^-2^: (a) surface topography images and their ablation temperature curves up to 1000 s; (b) linear ablation rate of the ZrC and ZrC-SiC coatings after oxyacetylene ablation at 2.4 MW m^-2^; (c) XRD patterns of the ZrC and ZrC-SiC coatings after oxyacetylene ablation at 2.4 MW m^-2^; (d) surface image of the ZrC-SiC coating after oxyacetylene ablation at 2.4 MW m^-2^ for 3080 s, which exhibited typical structure of twins; (e) EDS maps of the HAADF image of the twins.

Figure S5. Characterization of the samples under Ar-H_2_ plasma ablation: (a) surface topography images of the ZrC and ZrC-SiC coating, and their ablation temperature curves; (b-c) cross-section images of the (b) ZrC coating and (c) ZrC-SiC coating after plasma ablation; (d-e) 3D surface topography images of the (d) ZrC coating and (e) ZrC-SiC coating after plasma ablation; (f) surface image and (g) its EDS maps of the ZrC-SiC coating after plasma ablation for 600 s.

Figure S6. (a-b) cross-section image of the HfC-SiC coating; (c) dispersed SiC phase and its EDS maps; (d)XRD pattern of the HfC-SiC coating.

Figure S7. (a) cross-section image of the ablation center of the ZrC-SiC coating; (b) schematic diagram of the ablation behavior; (c) EPMA results of the ceramic phases in the C/C-ZrC-SiC sample when exposed to oxygen.

Table S1. Comparison of linear ablation rate, ablation time and ablation temperature of the thermal protection systems based on C/C under oxyacetylene torch ablation conditions.

| Protection Systems | temperature | time | LAR | references |
| --- | --- | --- | --- | --- |
| C/C-ZrC-SiC/ZrC-SiC | 2200 | 3080 | -0.075 | This work |
| Al_2_O_3_-SiO_2_-SiC coating | 1900 | 600 | 0.38 | [1] |
| (Hf-Z-Ti)C Coating | 2180 | 480 | -0.11 | [2] |
| Cf/ZrB2-SiC composites | 2570 | 600 | -0.186 | [3] |
| C/C-HfC-zRc-TaC-SiC composites | 2100 | 1000 | 0.701 | [4] |
| C/C-ZrC-SiC composites | 2600 | 1520 | -0.53 | [5] |
| Zr_0.92_Ta_0.04_Si_0.04_C coating | 1950 | 900 | -0.1 | [6] |
| Zr_0.92_Si_0.08_**C** coating | 1950 | 900 | -3.4 | [6] |
| C/C-HfC-ZrC composites | 2500 | 600 | 0.107 | [7] |
| C/C-HfC-SiC composites | 2500 | 500 | 0.297 | [8] |
| C/C-(Hf_0.5_Zr_0.3_Ti_0.2_)C-W-Cu composites | 2600 | 300 | -0.193 | [9] |
| Cf/SiC-ZrB_2_-ZrC composites | 1850 | 1000 | 1.3 | [10] |
| YSi_2_-doped ZrC-SiC-ZrSi_2_ coating | 2000 | 120 | -2.5 | [11] |
| (Hf_0.4_Zr_0.4_Ta_0.2_)B_2_-SiC-Si coating | 2300 | 900 | 0.4 | [12] |
| C_f_-HfC_0.76_N_0.24_ composites | 3000 | 900 | 0.7 | [13] |
| C/C-(Hf,Zr,Ti)C composites | 2500 | 240 | -1.86 | [14] |
| Y_2_O_3_ reinforced Cf/HfB_2_-SiC | 2700 | 300 | 0.67 | [15] |
| Gradient ZrC-SiC multilayer coating | 2000 | 600 | -0.37 | [16] |

Table S2. Parameters of the plasma spraying process for three coatings.

| Parameters | values |
| --- | --- |
| Spraying power, kW | 42-46 |
| Primary gas Ar, slpm | 70 |
| Carrier gas Ar, slpm | 5 |
| Second gas H_2_, slpm | 2.0-2.5 |
| Powder feed rate, g/min | ~20 |
| Spraying distance, mm | 100 |

**Table S3.** Material physical and thermal properties used in the present simulation process.

| Materials | *ρ* (g/cm^3^) | *α* (×10^−6^K^−1^) | *λ* (W/m·^o^C) | *E* (GPa) | *C* (J/(kg·K)) | *ν* |
| --- | --- | --- | --- | --- | --- | --- |
| C/C-ZrC-SiC *^a^* | 3.1 | 3.5 | 44 | 200 | 1700 | 0.27 |
| ZrC *^b^* | 6.71 | 6.7 | 20.5 | 380 | 325-354 | 0.2 |
| ZrC-20wt. %SiC *^c^* | 5.51 | 5.95 | 54.65 | 190 | 410 | 0.19 |
| ZrO_2_ *^b^* | 5.65 | 10 | 2.3 | 200 | 420 | 0.3 |

*^a^* experimentally measured data; *^b^* data retrieved from the database, such as [*www.matweb.com*](http://www.matweb.com); *^c^* data calculated using the rule of mixtures.

References

[1] Yan M, Hu C, Li J, Zhao R, Pang S, Liang B, et al. An Unusual Carbon–Ceramic Composite with Gradients in Composition and Porosity Delivering Outstanding Thermal Protection Performance up to 1900 °C. Advanced Functional Materials 2022;32(36):2204133.

[2] Li J, Zhang Y, Fu Y, Li T, Zhang J, Yang D, et al. Twin Toughening‐Driven Martensitic Transformation Strategy Synergistic Improvement for Plasticity‐Thermal Shock Resistance of (Hf-Zr-Ti)C Ceramic Coating in Severe Thermal Environments. Advanced Science 2025;12(26):2503226.

[3] Zhang C, Hu P, Xun L, Zhou Y, Han J, Zhang X. A universal strategy towards the fabrication of ultra-high temperature ceramic matrix composites with outstanding mechanical properties and ablation resistance. Composites Part B: Engineering 2024;280:111485.

[4] Zhang M, Liu T, Hu D, Fu Q. Ablation behavior of UHTCs carbide-modified C/C composites in extreme aerobic environments (3000 ℃): Evolution mechanisms of oxides film structure. Corrosion Science 2025;253:113036.

[5] Wang R, Cheng C, Yang X, Bai L, Zhang J, Fei J, et al. Ablation behavior of C/C-ZrC-SiC composites under oxyacetylene, plasma, and plasma-solid particle environments: A comparative investigation. Materials Characterization 2025;219:114649.

[6] Hu D, Li X, Chen S, Zhang Y, Fan Z, Yan K, et al. Design of long-term ablation protective Zr_0.92_Ta_0.04_Si_0.04_O_2_ coating via large lattice distortion and limited phase precipitation. Corrosion Science 2025;245:112689.

[7] Dong Z, Yu H, Zhang Y, Liu B, Zhang H, Zhang J, et al. C/C-HfC-ZrC composites with excellent long-term ablation performance prepared by combining PIP and RMI. Ceramics International 2024;50(8):13912-13923.

[8] Zhao R, Pang S, liang B, Li J, Hu C, Tang S, et al. Comparative ablation behaviors of C/SiC–ZrC and C/SiC–HfC composites prepared by ceramization of carbon aerogel preforms. Corrosion Science 2023;225:111623.

[9] Xu J, Sun W, Xiong X, Zhang H. C/C-(Hf_0.5_Zr_0.3_Ti_0.2_)C–W–Cu composites: Long-term ablation resistance based on active-passive protection at 2600  C. Composites Part B: Engineering 2025;289:111889.

[10] Hu C, Pang S, Tang S, Wang S, Huang H, Cheng H-M. Ablation and mechanical behavior of a sandwich-structured composite with an inner layer of Cf/SiC between two outer layers of Cf/SiC–ZrB_2_–ZrC. Corrosion Science 2014;80:154-163.

[11] Ma J, Kou S, Yang S, Liu Y, Luan C, Wang P, et al. Ablation performance of C/C-ZrC-SiC composites with in-situ YSi_2_-doped ZrC-SiC-ZrSi_2_ coating under oxyacetylene torch. Corrosion Science 2022;209:110802.

[12] Jiang Y, Hu C, Liang B, Pang S, Li J, Tang S. Cyclic ablation resistance at 2300 °C of (Hf_0.4_Zr_0.4_Ta_0.2_)B_2_-SiC-Si coating for C/SiC composites prepared by SiC-assisted reactive infiltration of silicon. Surface and Coatings Technology 2022;451:129072.

[13] Peng Z, Ma Q, Cui Y, Chen S, Cao F, Xiong X. Tailored Csf/HfC_0.76_N_0.24_ composites for superior ablation resistance at 3000 °C. Advanced Powder Materials 2025;4(2):100281.

[14] Gao S, Wang Y, Hu J, Zeng Y, Xiong X. Ablation behaviour of multi-component (Hf, Zr, Ti)C ceramics modified carbon/carbon composites. Journal of the European Ceramic Society 2025;45(5):117141.

[15] Zhang F, Ni D, Chen B, Cai F, Wang H, Gao L, et al. Long term ablation behavior and mechanisms of Y_2_O_3_ reinforced Cf/HfB2-SiC composites. Ceramics International 2025;51(13):18296-18304.

[16] Feng G, Li H, Yao X, Ren B, Jia Y, Sun J. Evaluation of ablation resistant ZrC-SiC multilayer coating for SiC coated carbon/carbon composites under oxyacetylene and laser conditions. Corrosion Science 2022;205:110427.
